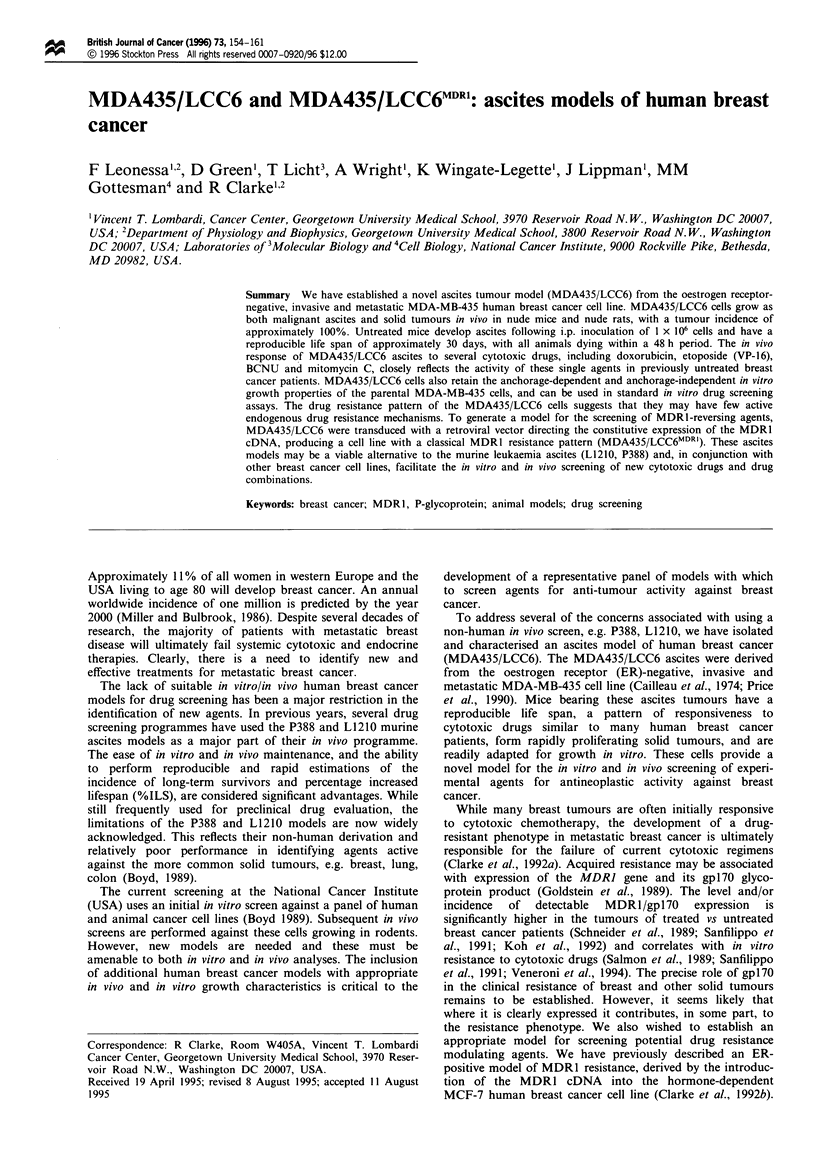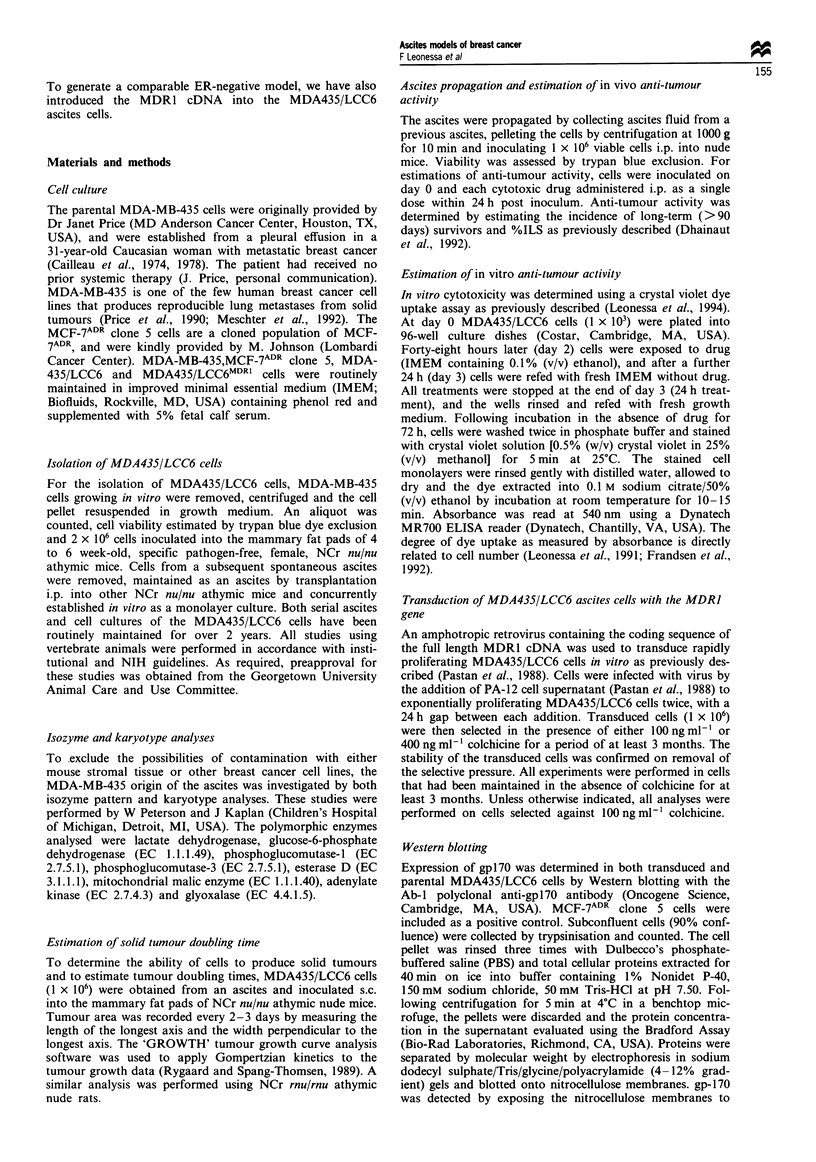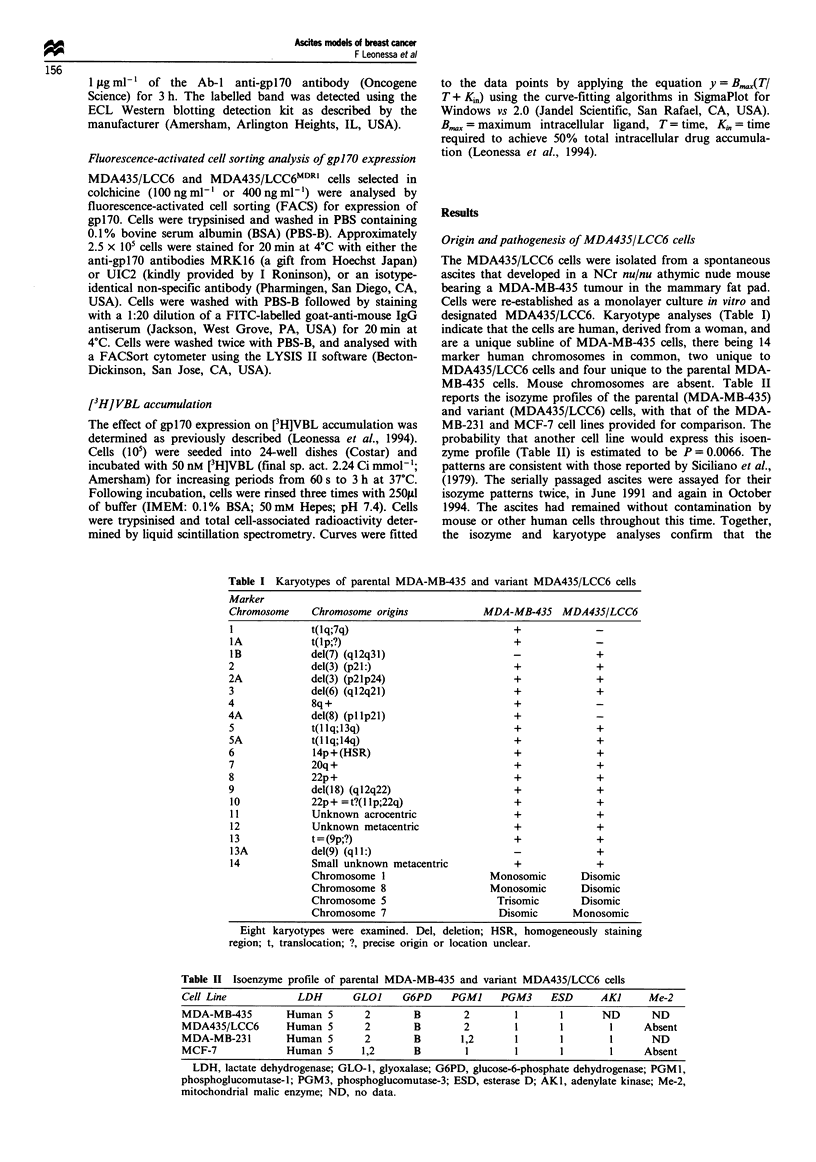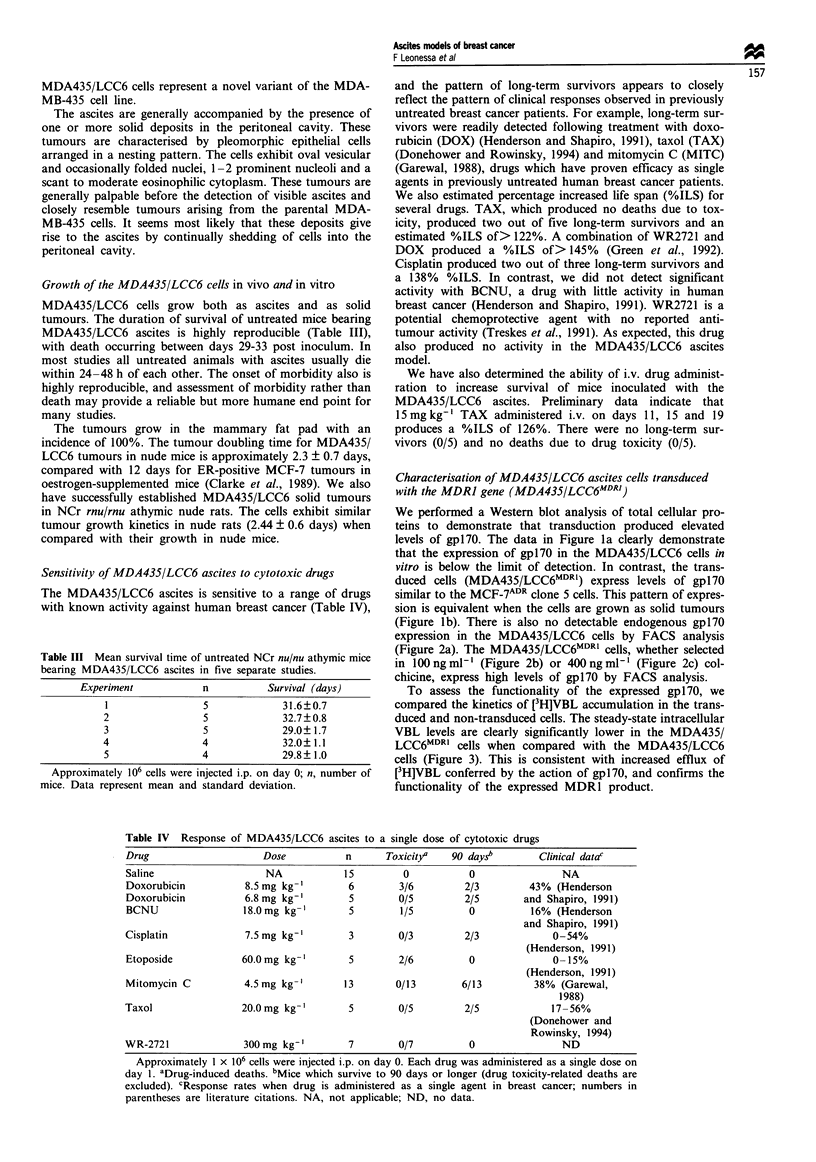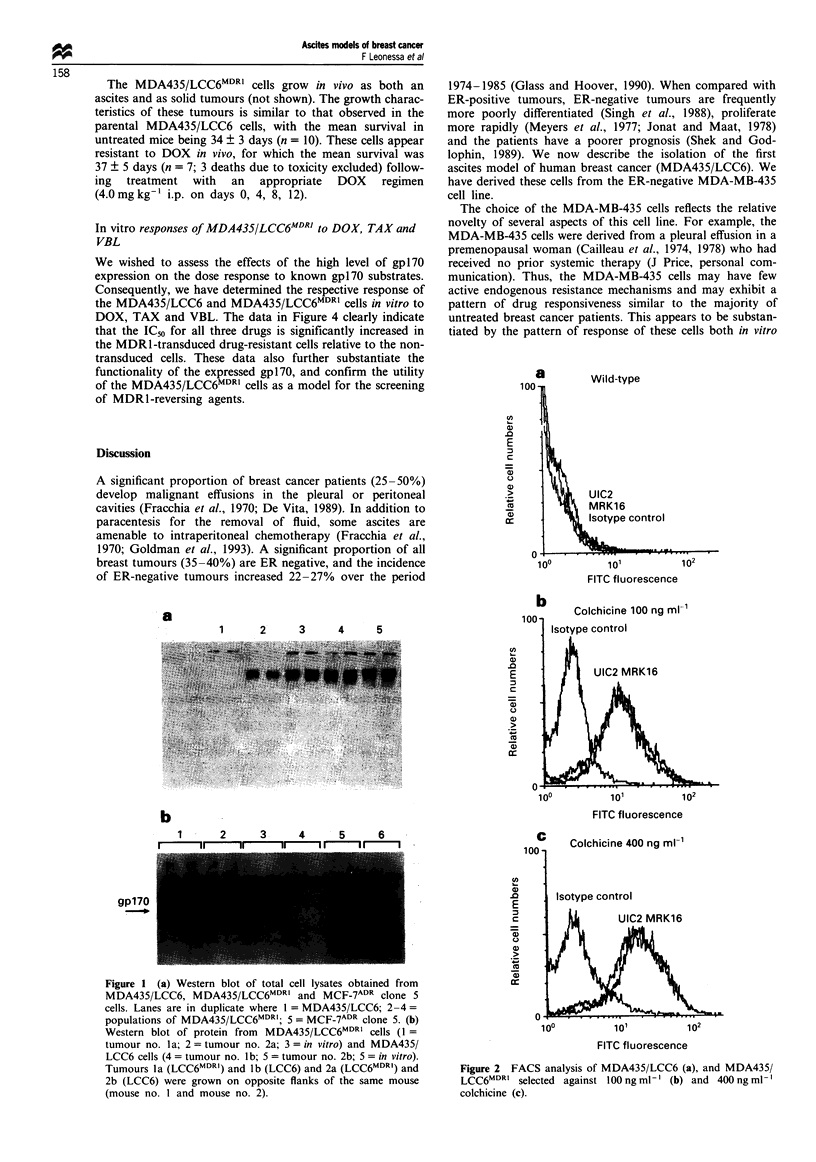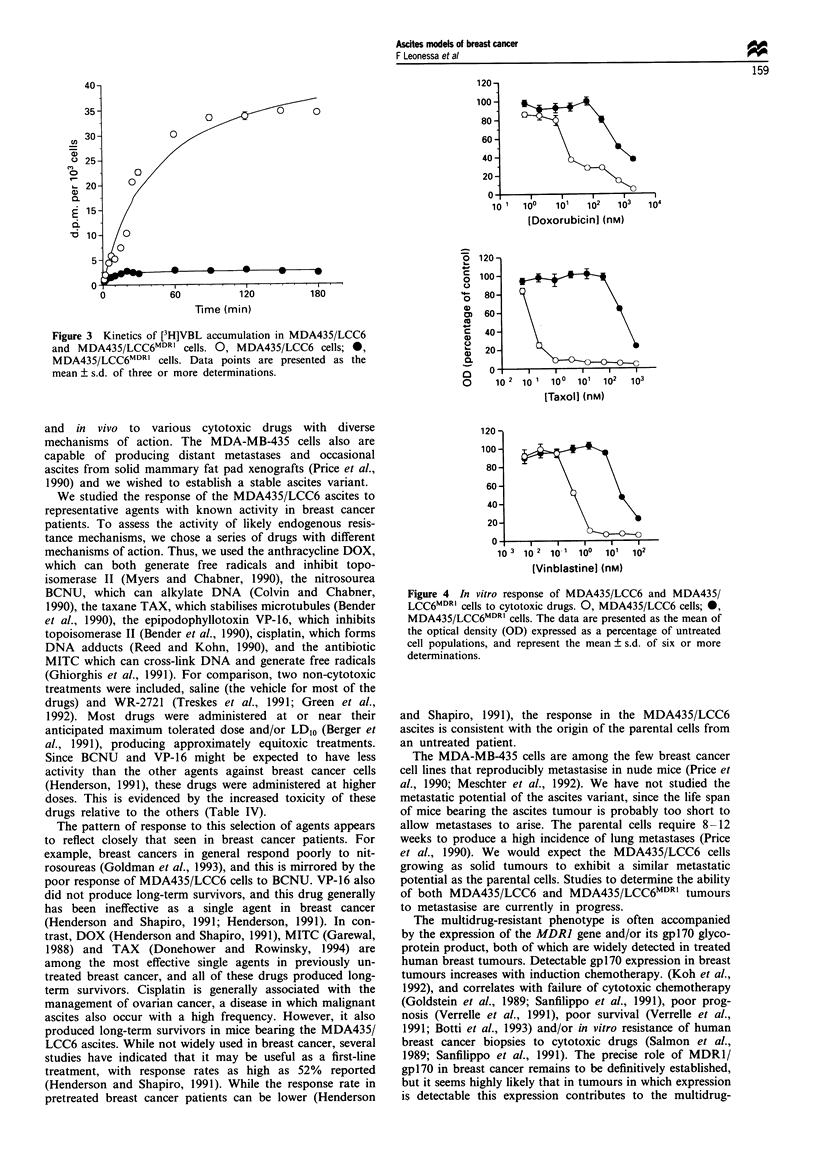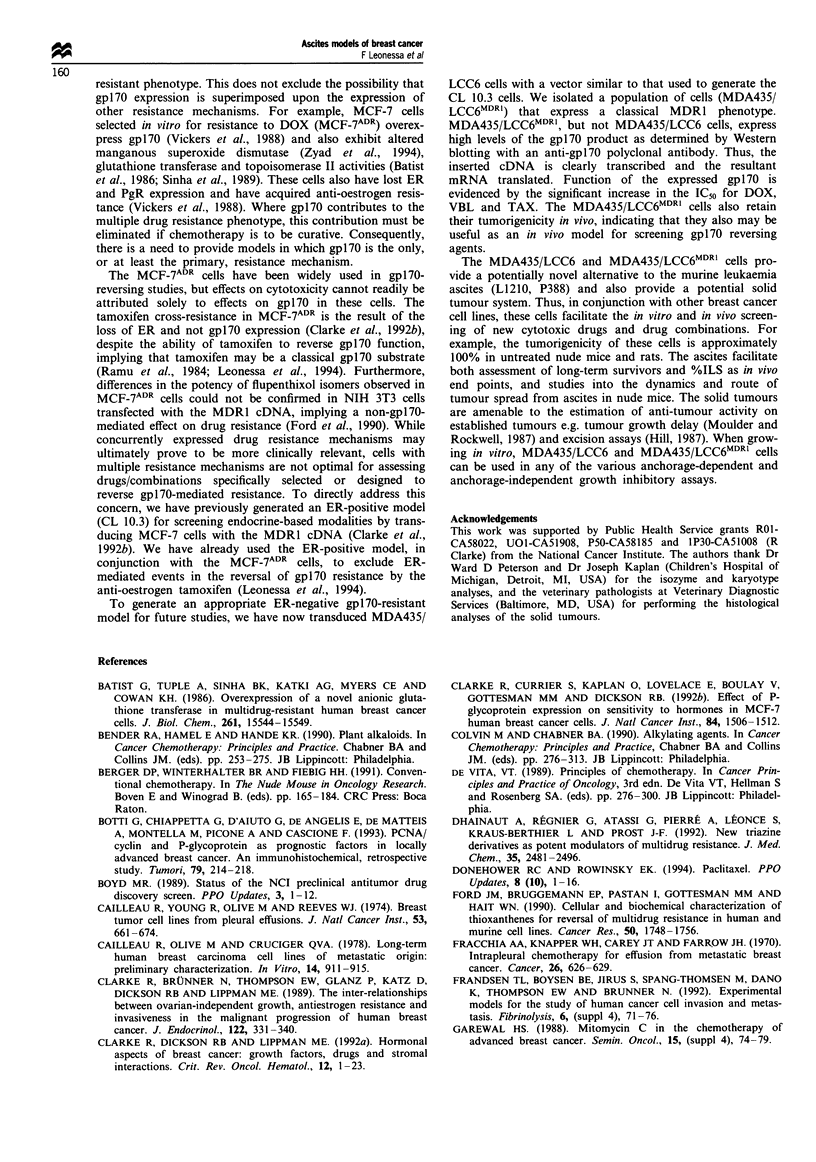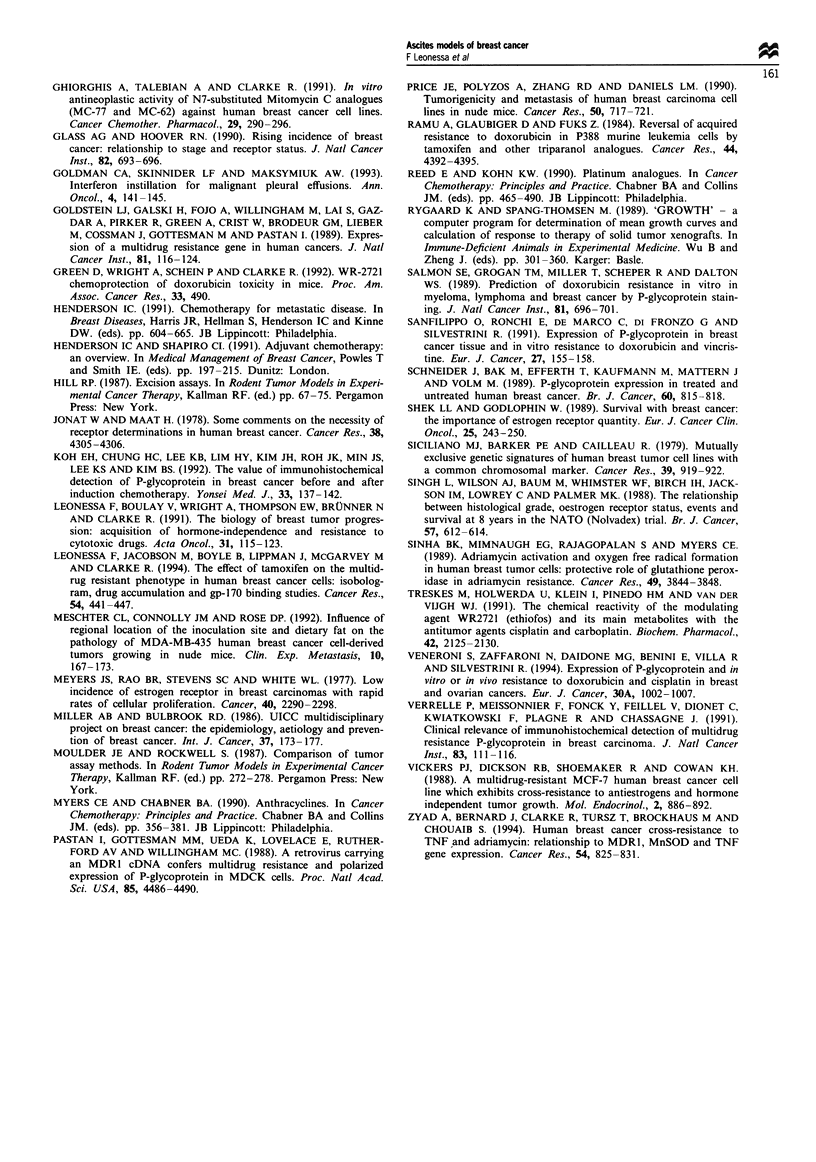# MDA435/LCC6 and MDA435/LCC6MDR1: ascites models of human breast cancer.

**DOI:** 10.1038/bjc.1996.29

**Published:** 1996-01

**Authors:** F. Leonessa, D. Green, T. Licht, A. Wright, K. Wingate-Legette, J. Lippman, M. M. Gottesman, R. Clarke

**Affiliations:** Vincent T. Lombardi, Cancer Center, Georgetown University Medical School, Washington DC 20007, USA.

## Abstract

**Images:**


British Journal of Cancer (1996) 73, 154-161
© 1996 Stockton Press All rights reserved 0007-0920/96 $12.00

### MDA435/LCC6 and MDA435/LCC6$^{MDR1}$: ascites models of human breast cancer

F Leonessa[1,2], D Green[1], T Licht[3], A Wright[1], K Wingate-Legette[1], J Lippman[1], MM Gottesman[4] and R Clarke[1,2]

[1]*Vincent T. Lombardi, Cancer Center, Georgetown University Medical School, 3970 Reservoir Road N.W., Washington DC 20007, USA;* [2]*Department of Physiology and Biophysics, Georgetown University Medical School, 3800 Reservoir Road N.W., Washington DC 20007, USA; Laboratories of* [3]*Molecular Biology and* [4]*Cell Biology, National Cancer Institute, 9000 Rockville Pike, Bethesda, MD 20982, USA.*

**Summary** We have established a novel ascites tumour model (MDA435/LCC6) from the oestrogen receptor-negative, invasive and metastatic MDA-MB-435 human breast cancer cell line. MDA435/LCC6 cells grow as both malignant ascites and solid tumours *in vivo* in nude mice and nude rats, with a tumour incidence of approximately 100%. Untreated mice develop ascites following i.p. inoculation of $1 \times 10^6$ cells and have a reproducible life span of approximately 30 days, with all animals dying within a 48 h period. The *in vivo* response of MDA435/LCC6 ascites to several cytotoxic drugs, including doxorubicin, etoposide (VP-16), BCNU and mitomycin C, closely reflects the activity of these single agents in previously untreated breast cancer patients. MDA435/LCC6 cells also retain the anchorage-dependent and anchorage-independent *in vitro* growth properties of the parental MDA-MB-435 cells, and can be used in standard *in vitro* drug screening assays. The drug resistance pattern of the MDA435/LCC6 cells suggests that they may have few active endogenous drug resistance mechanisms. To generate a model for the screening of MDR1-reversing agents, MDA435/LCC6 were transduced with a retroviral vector directing the constitutive expression of the MDR1 cDNA, producing a cell line with a classical MDR1 resistance pattern (MDA435/LCC6$^{MDR1}$). These ascites models may be a viable alternative to the murine leukaemia ascites (L1210, P388) and, in conjunction with other breast cancer cell lines, facilitate the *in vitro* and *in vivo* screening of new cytotoxic drugs and drug combinations.

**Keywords:** breast cancer; MDR1, P-glycoprotein; animal models; drug screening

Approximately 11% of all women in western Europe and the USA living to age 80 will develop breast cancer. An annual worldwide incidence of one million is predicted by the year 2000 (Miller and Bulbrook, 1986). Despite several decades of research, the majority of patients with metastatic breast disease will ultimately fail systemic cytotoxic and endocrine therapies. Clearly, there is a need to identify new and effective treatments for metastatic breast cancer.

The lack of suitable *in vitro/in vivo* human breast cancer models for drug screening has been a major restriction in the identification of new agents. In previous years, several drug screening programmes have used the P388 and L1210 murine ascites models as a major part of their *in vivo* programme. The ease of *in vitro* and *in vivo* maintenance, and the ability to perform reproducible and rapid estimations of the incidence of long-term survivors and percentage increased lifespan (%ILS), are considered significant advantages. While still frequently used for preclinical drug evaluation, the limitations of the P388 and L1210 models are now widely acknowledged. This reflects their non-human derivation and relatively poor performance in identifying agents active against the more common solid tumours, e.g. breast, lung, colon (Boyd, 1989).

The current screening at the National Cancer Institute (USA) uses an initial *in vitro* screen against a panel of human and animal cancer cell lines (Boyd 1989). Subsequent *in vivo* screens are performed against these cells growing in rodents. However, new models are needed and these must be amenable to both *in vitro* and *in vivo* analyses. The inclusion of additional human breast cancer models with appropriate *in vivo* and *in vitro* growth characteristics is critical to the development of a representative panel of models with which to screen agents for anti-tumour activity against breast cancer.

To address several of the concerns associated with using a non-human *in vivo* screen, e.g. P388, L1210, we have isolated and characterised an ascites model of human breast cancer (MDA435/LCC6). The MDA435/LCC6 ascites were derived from the oestrogen receptor (ER)-negative, invasive and metastatic MDA-MB-435 cell line (Cailleau *et al.*, 1974; Price *et al.*, 1990). Mice bearing these ascites tumours have a reproducible life span, a pattern of responsiveness to cytotoxic drugs similar to many human breast cancer patients, form rapidly proliferating solid tumours, and are readily adapted for growth *in vitro*. These cells provide a novel model for the *in vitro* and *in vivo* screening of experimental agents for antineoplastic activity against breast cancer.

While many breast tumours are often initially responsive to cytotoxic chemotherapy, the development of a drug-resistant phenotype in metastatic breast cancer is ultimately responsible for the failure of current cytotoxic regimens (Clarke *et al.*, 1992*a*). Acquired resistance may be associated with expression of the *MDR1* gene and its gp170 glycoprotein product (Goldstein *et al.*, 1989). The level and/or incidence of detectable MDR1/gp170 expression is significantly higher in the tumours of treated *vs* untreated breast cancer patients (Schneider *et al.*, 1989; Sanfilippo *et al.*, 1991; Koh *et al.*, 1992) and correlates with *in vitro* resistance to cytotoxic drugs (Salmon *et al.*, 1989; Sanfilippo *et al.*, 1991; Veneroni *et al.*, 1994). The precise role of gp170 in the clinical resistance of breast and other solid tumours remains to be established. However, it seems likely that where it is clearly expressed it contributes, in some part, to the resistance phenotype. We also wished to establish an appropriate model for screening potential drug resistance modulating agents. We have previously described an ER-positive model of MDR1 resistance, derived by the introduction of the MDR1 cDNA into the hormone-dependent MCF-7 human breast cancer cell line (Clarke *et al.*, 1992*b*).

Correspondence: R Clarke, Room W405A, Vincent T. Lombardi Cancer Center, Georgetown University Medical School, 3970 Reservoir Road N.W., Washington DC 20007, USA.
Received 19 April 1995; revised 8 August 1995; accepted 11 August 1995

To generate a comparable ER-negative model, we have also introduced the MDR1 cDNA into the MDA435/LCC6 ascites cells.

### Materials and methods

### *Cell culture*

The parental MDA-MB-435 cells were originally provided by Dr Janet Price (MD Anderson Cancer Center, Houston, TX, USA), and were established from a pleural effusion in a 31-year-old Caucasian woman with metastatic breast cancer (Cailleau *et al.*, 1974, 1978). The patient had received no prior systemic therapy (J. Price, personal communication). MDA-MB-435 is one of the few human breast cancer cell lines that produces reproducible lung metastases from solid tumours (Price *et al.*, 1990; Meschter *et al.*, 1992). The MCF-$7^{ADR}$ clone 5 cells are a cloned population of MCF-$7^{ADR}$, and were kindly provided by M. Johnson (Lombardi Cancer Center). MDA-MB-435,MCF-$7^{ADR}$ clone 5, MDA-435/LCC6 and MDA435/$LCC6^{MDR1}$ cells were routinely maintained in improved minimal essential medium (IMEM; Biofluids, Rockville, MD, USA) containing phenol red and supplemented with 5% fetal calf serum.

### *Isolation of MDA435/LCC6 cells*

For the isolation of MDA435/LCC6 cells, MDA-MB-435 cells growing *in vitro* were removed, centrifuged and the cell pellet resuspended in growth medium. An aliquot was counted, cell viability estimated by trypan blue dye exclusion and $2 \times 10^6$ cells inoculated into the mammary fat pads of 4 to 6 week-old, specific pathogen-free, female, NCr *nu/nu* athymic mice. Cells from a subsequent spontaneous ascites were removed, maintained as an ascites by transplantation i.p. into other NCr *nu/nu* athymic mice and concurrently established *in vitro* as a monolayer culture. Both serial ascites and cell cultures of the MDA435/LCC6 cells have been routinely maintained for over 2 years. All studies using vertebrate animals were performed in accordance with institutional and NIH guidelines. As required, preapproval for these studies was obtained from the Georgetown University Animal Care and Use Committee.

### *Isozyme and karyotype analyses*

To exclude the possibilities of contamination with either mouse stromal tissue or other breast cancer cell lines, the MDA-MB-435 origin of the ascites was investigated by both isozyme pattern and karyotype analyses. These studies were performed by W Peterson and J Kaplan (Children's Hospital of Michigan, Detroit, MI, USA). The polymorphic enzymes analysed were lactate dehydrogenase, glucose-6-phosphate dehydrogenase (EC 1.1.1.49), phosphoglucomutase-1 (EC 2.7.5.1), phosphoglucomutase-3 (EC 2.7.5.1), esterase D (EC 3.1.1.1), mitochondrial malic enzyme (EC 1.1.1.40), adenylate kinase (EC 2.7.4.3) and glyoxalase (EC 4.4.1.5).

### *Estimation of solid tumour doubling time*

To determine the ability of cells to produce solid tumours and to estimate tumour doubling times, MDA435/LCC6 cells ($1 \times 10^6$) were obtained from an ascites and inoculated s.c. into the mammary fat pads of NCr *nu/nu* athymic nude mice. Tumour area was recorded every 2–3 days by measuring the length of the longest axis and the width perpendicular to the longest axis. The 'GROWTH' tumour growth curve analysis software was used to apply Gompertzian kinetics to the tumour growth data (Rygaard and Spang-Thomsen, 1989). A similar analysis was performed using NCr *rnu/rnu* athymic nude rats.

### *Ascites propagation and estimation of* in vivo *anti-tumour activity*

The ascites were propagated by collecting ascites fluid from a previous ascites, pelleting the cells by centrifugation at 1000 **g** for 10 min and inoculating $1 \times 10^6$ viable cells i.p. into nude mice. Viability was assessed by trypan blue exclusion. For estimations of anti-tumour activity, cells were inoculated on day 0 and each cytotoxic drug administered i.p. as a single dose within 24 h post inoculum. Anti-tumour activity was determined by estimating the incidence of long-term (>90 days) survivors and %ILS as previously described (Dhainaut *et al.*, 1992).

### *Estimation of* in vitro *anti-tumour activity*

*In vitro* cytotoxicity was determined using a crystal violet dye uptake assay as previously described (Leonessa *et al.*, 1994). At day 0 MDA435/LCC6 cells ($1 \times 10^3$) were plated into 96-well culture dishes (Costar, Cambridge, MA, USA). Forty-eight hours later (day 2) cells were exposed to drug (IMEM containing 0.1% (v/v) ethanol), and after a further 24 h (day 3) cells were refed with fresh IMEM without drug. All treatments were stopped at the end of day 3 (24 h treatment), and the wells rinsed and refed with fresh growth medium. Following incubation in the absence of drug for 72 h, cells were washed twice in phosphate buffer and stained with crystal violet solution [0.5% (w/v) crystal violet in 25% (v/v) methanol] for 5 min at 25°C. The stained cell monolayers were rinsed gently with distilled water, allowed to dry and the dye extracted into 0.1 M sodium citrate/50% (v/v) ethanol by incubation at room temperature for 10–15 min. Absorbance was read at 540 nm using a Dynatech MR700 ELISA reader (Dynatech, Chantilly, VA, USA). The degree of dye uptake as measured by absorbance is directly related to cell number (Leonessa *et al.*, 1991; Frandsen *et al.*, 1992).

### *Transduction of MDA435/LCC6 ascites cells with the MDR1 gene*

An amphotropic retrovirus containing the coding sequence of the full length MDR1 cDNA was used to transduce rapidly proliferating MDA435/LCC6 cells *in vitro* as previously described (Pastan *et al.*, 1988). Cells were infected with virus by the addition of PA-12 cell supernatant (Pastan *et al.*, 1988) to exponentially proliferating MDA435/LCC6 cells twice, with a 24 h gap between each addition. Transduced cells ($1 \times 10^6$) were then selected in the presence of either 100 ng $ml^{-1}$ or 400 ng $ml^{-1}$ colchicine for a period of at least 3 months. The stability of the transduced cells was confirmed on removal of the selective pressure. All experiments were performed in cells that had been maintained in the absence of colchicine for at least 3 months. Unless otherwise indicated, all analyses were performed on cells selected against 100 ng $ml^{-1}$ colchicine.

### *Western blotting*

Expression of gp170 was determined in both transduced and parental MDA435/LCC6 cells by Western blotting with the Ab-1 polyclonal anti-gp170 antibody (Oncogene Science, Cambridge, MA, USA). MCF-$7^{ADR}$ clone 5 cells were included as a positive control. Subconfluent cells (90% confluence) were collected by trypsinisation and counted. The cell pellet was rinsed three times with Dulbecco's phosphate-buffered saline (PBS) and total cellular proteins extracted for 40 min on ice into buffer containing 1% Nonidet P-40, 150 mM sodium chloride, 50 mM Tris-HCl at pH 7.50. Following centrifugation for 5 min at 4°C in a benchtop microfuge, the pellets were discarded and the protein concentration in the supernatant evaluated using the Bradford Assay (Bio-Rad Laboratories, Richmond, CA, USA). Proteins were separated by molecular weight by electrophoresis in sodium dodecyl sulphate/Tris/glycine/polyacrylamide (4–12% gradient) gels and blotted onto nitrocellulose membranes. gp-170 was detected by exposing the nitrocellulose membranes to

1 μg ml$^{-1}$ of the Ab-1 anti-gp170 antibody (Oncogene Science) for 3 h. The labelled band was detected using the ECL Western blotting detection kit as described by the manufacturer (Amersham, Arlington Heights, IL, USA).

*Fluorescence-activated cell sorting analysis of gp170 expression*

MDA435/LCC6 and MDA435/LCC6$^{MDR1}$ cells selected in colchicine (100 ng ml$^{-1}$ or 400 ng ml$^{-1}$) were analysed by fluorescence-activated cell sorting (FACS) for expression of gp170. Cells were trypsinised and washed in PBS containing 0.1% bovine serum albumin (BSA) (PBS-B). Approximately $2.5 \times 10^5$ cells were stained for 20 min at 4°C with either the anti-gp170 antibodies MRK16 (a gift from Hoechst Japan) or UIC2 (kindly provided by I Roninson), or an isotype-identical non-specific antibody (Pharmingen, San Diego, CA, USA). Cells were washed with PBS-B followed by staining with a 1:20 dilution of a FITC-labelled goat-anti-mouse IgG antiserum (Jackson, West Grove, PA, USA) for 20 min at 4°C. Cells were washed twice with PBS-B, and analysed with a FACSort cytometer using the LYSIS II software (Becton-Dickinson, San Jose, CA, USA).

*[$^3$H]VBL accumulation*

The effect of gp170 expression on [$^3$H]VBL accumulation was determined as previously described (Leonessa *et al.*, 1994). Cells ($10^5$) were seeded into 24-well dishes (Costar) and incubated with 50 nM [$^3$H]VBL (final sp. act. 2.24 Ci mmol$^{-1}$; Amersham) for increasing periods from 60 s to 3 h at 37°C. Following incubation, cells were rinsed three times with 250μl of buffer (IMEM: 0.1% BSA; 50 mM Hepes; pH 7.4). Cells were trypsinised and total cell-associated radioactivity determined by liquid scintillation spectrometry. Curves were fitted to the data points by applying the equation $y = B_{max}(T/T + K_{in})$ using the curve-fitting algorithms in SigmaPlot for Windows *vs* 2.0 (Jandel Scientific, San Rafael, CA, USA). $B_{max}$ = maximum intracellular ligand, $T$ = time, $K_{in}$ = time required to achieve 50% total intracellular drug accumulation (Leonessa *et al.*, 1994).

### Results

*Origin and pathogenesis of MDA435/LCC6 cells*

The MDA435/LCC6 cells were isolated from a spontaneous ascites that developed in a NCr *nu/nu* athymic nude mouse bearing a MDA-MB-435 tumour in the mammary fat pad. Cells were re-established as a monolayer culture *in vitro* and designated MDA435/LCC6. Karyotype analyses (Table I) indicate that the cells are human, derived from a woman, and are a unique subline of MDA-MB-435 cells, there being 14 marker human chromosomes in common, two unique to MDA435/LCC6 cells and four unique to the parental MDA-MB-435 cells. Mouse chromosomes are absent. Table II reports the isozyme profiles of the parental (MDA-MB-435) and variant (MDA435/LCC6) cells, with that of the MDA-MB-231 and MCF-7 cell lines provided for comparison. The probability that another cell line would express this isoenzyme profile (Table II) is estimated to be $P = 0.0066$. The patterns are consistent with those reported by Siciliano *et al.*, (1979). The serially passaged ascites were assayed for their isozyme patterns twice, in June 1991 and again in October 1994. The ascites had remained without contamination by mouse or other human cells throughout this time. Together, the isozyme and karyotype analyses confirm that the

**Table I** Karyotypes of parental MDA-MB-435 and variant MDA435/LCC6 cells

| *Marker Chromosome* | *Chromosome origins* | *MDA-MB-435* | *MDA435/LCC6* |
|---|---|---|---|
| 1 | t(1q;7q) | + | – |
| 1A | t(1p;?) | + | – |
| 1B | del(7) (q12q31) | – | + |
| 2 | del(3) (p21:) | + | + |
| 2A | del(3) (p21p24) | + | + |
| 3 | del(6) (q12q21) | + | + |
| 4 | 8q+ | + | – |
| 4A | del(8) (p11p21) | + | – |
| 5 | t(11q;13q) | + | + |
| 5A | t(11q;14q) | + | + |
| 6 | 14p+(HSR) | + | + |
| 7 | 20q+ | + | + |
| 8 | 22p+ | + | + |
| 9 | del(18) (q12q22) | + | + |
| 10 | 22p+ = t?(11p;22q) | + | + |
| 11 | Unknown acrocentric | + | + |
| 12 | Unknown metacentric | + | + |
| 13 | t=(9p;?) | + | + |
| 13A | del(9) (q11:) | – | + |
| 14 | Small unknown metacentric | + | + |
| | Chromosome 1 | Monosomic | Disomic |
| | Chromosome 8 | Monosomic | Disomic |
| | Chromosome 5 | Trisomic | Disomic |
| | Chromosome 7 | Disomic | Monosomic |

Eight karyotypes were examined. Del, deletion; HSR, homogeneously staining region; t, translocation; ?, precise origin or location unclear.

**Table II** Isoenzyme profile of parental MDA-MB-435 and variant MDA435/LCC6 cells

| *Cell Line* | *LDH* | *GLO1* | *G6PD* | *PGM1* | *PGM3* | *ESD* | *AK1* | *Me-2* |
|---|---|---|---|---|---|---|---|---|
| MDA-MB-435 | Human 5 | 2 | B | 2 | 1 | 1 | ND | ND |
| MDA435/LCC6 | Human 5 | 2 | B | 2 | 1 | 1 | 1 | Absent |
| MDA-MB-231 | Human 5 | 2 | B | 1,2 | 1 | 1 | 1 | ND |
| MCF-7 | Human 5 | 1,2 | B | 1 | 1 | 1 | 1 | Absent |

LDH, lactate dehydrogenase; GLO-1, glyoxalase; G6PD, glucose-6-phosphate dehydrogenase; PGM1, phosphoglucomutase-1; PGM3, phosphoglucomutase-3; ESD, esterase D; AK1, adenylate kinase; Me-2, mitochondrial malic enzyme; ND, no data.

MDA435/LCC6 cells represent a novel variant of the MDA-MB-435 cell line.

The ascites are generally accompanied by the presence of one or more solid deposits in the peritoneal cavity. These tumours are characterised by pleomorphic epithelial cells arranged in a nesting pattern. The cells exhibit oval vesicular and occasionally folded nuclei, 1–2 prominent nucleoli and a scant to moderate eosinophilic cytoplasm. These tumours are generally palpable before the detection of visible ascites and closely resemble tumours arising from the parental MDA-MB-435 cells. It seems most likely that these deposits give rise to the ascites by continually shedding of cells into the peritoneal cavity.

*Growth of the MDA435/LCC6 cells* in vivo *and* in vitro

MDA435/LCC6 cells grow both as ascites and as solid tumours. The duration of survival of untreated mice bearing MDA435/LCC6 ascites is highly reproducible (Table III), with death occurring between days 29-33 post inoculum. In most studies all untreated animals with ascites usually die within 24–48 h of each other. The onset of morbidity also is highly reproducible, and assessment of morbidity rather than death may provide a reliable but more humane end point for many studies.

The tumours grow in the mammary fat pad with an incidence of 100%. The tumour doubling time for MDA435/LCC6 tumours in nude mice is approximately 2.3 ± 0.7 days, compared with 12 days for ER-positive MCF-7 tumours in oestrogen-supplemented mice (Clarke *et al.*, 1989). We also have successfully established MDA435/LCC6 solid tumours in NCr *rnu/rnu* athymic nude rats. The cells exhibit similar tumour growth kinetics in nude rats (2.44 ± 0.6 days) when compared with their growth in nude mice.

*Sensitivity of MDA435/LCC6 ascites to cytotoxic drugs*

The MDA435/LCC6 ascites is sensitive to a range of drugs with known activity against human breast cancer (Table IV), and the pattern of long-term survivors appears to closely reflect the pattern of clinical responses observed in previously untreated breast cancer patients. For example, long-term survivors were readily detected following treatment with doxorubicin (DOX) (Henderson and Shapiro, 1991), taxol (TAX) (Donehower and Rowinsky, 1994) and mitomycin C (MITC) (Garewal, 1988), drugs which have proven efficacy as single agents in previously untreated human breast cancer patients. We also estimated percentage increased life span (%ILS) for several drugs. TAX, which produced no deaths due to toxicity, produced two out of five long-term survivors and an estimated %ILS of > 122%. A combination of WR2721 and DOX produced a %ILS of > 145% (Green *et al.*, 1992). Cisplatin produced two out of three long-term survivors and a 138% %ILS. In contrast, we did not detect significant activity with BCNU, a drug with little activity in human breast cancer (Henderson and Shapiro, 1991). WR2721 is a potential chemoprotective agent with no reported antitumour activity (Treskes *et al.*, 1991). As expected, this drug also produced no activity in the MDA435/LCC6 ascites model.

We have also determined the ability of i.v. drug administration to increase survival of mice inoculated with the MDA435/LCC6 ascites. Preliminary data indicate that 15 mg $kg^{-1}$ TAX administered i.v. on days 11, 15 and 19 produces a %ILS of 126%. There were no long-term survivors (0/5) and no deaths due to drug toxicity (0/5).

*Characterisation of MDA435/LCC6 ascites cells transduced with the MDR1 gene (MDA435/LCC6$^{MDR1}$)*

We performed a Western blot analysis of total cellular proteins to demonstrate that transduction produced elevated levels of gp170. The data in Figure 1a clearly demonstrate that the expression of gp170 in the MDA435/LCC6 cells *in vitro* is below the limit of detection. In contrast, the transduced cells (MDA435/LCC6$^{MDR1}$) express levels of gp170 similar to the MCF-7$^{ADR}$ clone 5 cells. This pattern of expression is equivalent when the cells are grown as solid tumours (Figure 1b). There is also no detectable endogenous gp170 expression in the MDA435/LCC6 cells by FACS analysis (Figure 2a). The MDA435/LCC6$^{MDR1}$ cells, whether selected in 100 ng $ml^{-1}$ (Figure 2b) or 400 ng $ml^{-1}$ (Figure 2c) colchicine, express high levels of gp170 by FACS analysis.

To assess the functionality of the expressed gp170, we compared the kinetics of [$^3$H]VBL accumulation in the transduced and non-transduced cells. The steady-state intracellular VBL levels are clearly significantly lower in the MDA435/LCC6$^{MDR1}$ cells when compared with the MDA435/LCC6 cells (Figure 3). This is consistent with increased efflux of [$^3$H]VBL conferred by the action of gp170, and confirms the functionality of the expressed MDR1 product.

**Table III** Mean survival time of untreated NCr *nu/nu* athymic mice bearing MDA435/LCC6 ascites in five separate studies.

| *Experiment* | n | *Survival (days)* |
|---|---|---|
| 1 | 5 | 31.6±0.7 |
| 2 | 5 | 32.7±0.8 |
| 3 | 5 | 29.0±1.7 |
| 4 | 4 | 32.0±1.1 |
| 5 | 4 | 29.8±1.0 |

Approximately $10^6$ cells were injected i.p. on day 0; *n*, number of mice. Data represent mean and standard deviation.

**Table IV** Response of MDA435/LCC6 ascites to a single dose of cytotoxic drugs

| *Drug* | *Dose* | n | *Toxicity[a]* | *90 days[b]* | *Clinical data[c]* |
|---|---|---|---|---|---|
| Saline | NA | 15 | 0 | 0 | NA |
| Doxorubicin | 8.5 mg $kg^{-1}$ | 6 | 3/6 | 2/3 | 43% (Henderson |
| Doxorubicin | 6.8 mg $kg^{-1}$ | 5 | 0/5 | 2/5 | and Shapiro, 1991) |
| BCNU | 18.0 mg $kg^{-1}$ | 5 | 1/5 | 0 | 16% (Henderson and Shapiro, 1991) |
| Cisplatin | 7.5 mg $kg^{-1}$ | 3 | 0/3 | 2/3 | 0–54% (Henderson, 1991) |
| Etoposide | 60.0 mg $kg^{-1}$ | 5 | 2/6 | 0 | 0–15% (Henderson, 1991) |
| Mitomycin C | 4.5 mg $kg^{-1}$ | 13 | 0/13 | 6/13 | 38% (Garewal, 1988) |
| Taxol | 20.0 mg $kg^{-1}$ | 5 | 0/5 | 2/5 | 17–56% (Donehower and Rowinsky, 1994) |
| WR-2721 | 300 mg $kg^{-1}$ | 7 | 0/7 | 0 | ND |

Approximately 1 × $10^6$ cells were injected i.p. on day 0. Each drug was administered as a single dose on day 1. [a]Drug-induced deaths. [b]Mice which survive to 90 days or longer (drug toxicity-related deaths are excluded). [c]Response rates when drug is administered as a single agent in breast cancer; numbers in parentheses are literature citations. NA, not applicable; ND, no data.

The MDA435/LCC6$^{MDR1}$ cells grow *in vivo* as both an ascites and as solid tumours (not shown). The growth characteristics of these tumours is similar to that observed in the parental MDA435/LCC6 cells, with the mean survival in untreated mice being $34 \pm 3$ days ($n = 10$). These cells appear resistant to DOX *in vivo*, for which the mean survival was $37 \pm 5$ days ($n = 7$; 3 deaths due to toxicity excluded) following treatment with an appropriate DOX regimen (4.0 mg $kg^{-1}$ i.p. on days 0, 4, 8, 12).

### *In vitro responses of MDA435/LCC6$^{MDR1}$ to DOX, TAX and VBL*

We wished to assess the effects of the high level of gp170 expression on the dose response to known gp170 substrates. Consequently, we have determined the respective response of the MDA435/LCC6 and MDA435/LCC6$^{MDR1}$ cells *in vitro* to DOX, TAX and VBL. The data in Figure 4 clearly indicate that the $IC_{50}$ for all three drugs is significantly increased in the MDR1-transduced drug-resistant cells relative to the non-transduced cells. These data also further substantiate the functionality of the expressed gp170, and confirm the utility of the MDA435/LCC6$^{MDR1}$ cells as a model for the screening of MDR1-reversing agents.

### Discussion

A significant proportion of breast cancer patients (25–50%) develop malignant effusions in the pleural or peritoneal cavities (Fracchia *et al*., 1970; De Vita, 1989). In addition to paracentesis for the removal of fluid, some ascites are amenable to intraperitoneal chemotherapy (Fracchia *et al*., 1970; Goldman *et al*., 1993). A significant proportion of all breast tumours (35–40%) are ER negative, and the incidence of ER-negative tumours increased 22–27% over the period 1974–1985 (Glass and Hoover, 1990). When compared with ER-positive tumours, ER-negative tumours are frequently more poorly differentiated (Singh *et al*., 1988), proliferate more rapidly (Meyers *et al*., 1977; Jonat and Maat, 1978) and the patients have a poorer prognosis (Shek and Godlophin, 1989). We now describe the isolation of the first ascites model of human breast cancer (MDA435/LCC6). We have derived these cells from the ER-negative MDA-MB-435 cell line.

The choice of the MDA-MB-435 cells reflects the relative novelty of several aspects of this cell line. For example, the MDA-MB-435 cells were derived from a pleural effusion in a premenopausal woman (Cailleau *et al*., 1974, 1978) who had received no prior systemic therapy (J Price, personal communication). Thus, the MDA-MB-435 cells may have few active endogenous resistance mechanisms and may exhibit a pattern of drug responsiveness similar to the majority of untreated breast cancer patients. This appears to be substantiated by the pattern of response of these cells both *in vitro*

**Figure 1** (**a**) Western blot of total cell lysates obtained from MDA435/LCC6, MDA435/LCC6$^{MDR1}$ and MCF-7$^{ADR}$ clone 5 cells. Lanes are in duplicate where 1 = MDA435/LCC6; 2–4 = populations of MDA435/LCC6$^{MDR1}$; 5 = MCF-7$^{ADR}$ clone 5. (**b**) Western blot of protein from MDA435/LCC6$^{MDR1}$ cells (1 = tumour no. 1a; 2 = tumour no. 2a; 3 = *in vitro*) and MDA435/LCC6 cells (4 = tumour no. 1b; 5 = tumour no. 2b; 5 = *in vitro*). Tumours 1a (LCC6$^{MDR1}$) and 1b (LCC6) and 2a (LCC6$^{MDR1}$) and 2b (LCC6) were grown on opposite flanks of the same mouse (mouse no. 1 and mouse no. 2).

**Figure 2** FACS analysis of MDA435/LCC6 (**a**), and MDA435/LCC6$^{MDR1}$ selected against 100 ng $ml^{-1}$ (**b**) and 400 ng $ml^{-1}$ colchicine (**c**).

**Figure 3** Kinetics of [$^3$H]VBL accumulation in MDA435/LCC6 and MDA435/LCC6$^{MDR1}$ cells. O, MDA435/LCC6 cells; ●, MDA435/LCC6$^{MDR1}$ cells. Data points are presented as the mean ± s.d. of three or more determinations.

**Figure 4** *In vitro* response of MDA435/LCC6 and MDA435/LCC6$^{MDR1}$ cells to cytotoxic drugs. O, MDA435/LCC6 cells; ●, MDA435/LCC6$^{MDR1}$ cells. The data are presented as the mean of the optical density (OD) expressed as a percentage of untreated cell populations, and represent the mean ± s.d. of six or more determinations.

and *in vivo* to various cytotoxic drugs with diverse mechanisms of action. The MDA-MB-435 cells also are capable of producing distant metastases and occasional ascites from solid mammary fat pad xenografts (Price *et al.*, 1990) and we wished to establish a stable ascites variant.

We studied the response of the MDA435/LCC6 ascites to representative agents with known activity in breast cancer patients. To assess the activity of likely endogenous resistance mechanisms, we chose a series of drugs with different mechanisms of action. Thus, we used the anthracycline DOX, which can both generate free radicals and inhibit topoisomerase II (Myers and Chabner, 1990), the nitrosourea BCNU, which can alkylate DNA (Colvin and Chabner, 1990), the taxane TAX, which stabilises microtubules (Bender *et al.*, 1990), the epipodophyllotoxin VP-16, which inhibits topoisomerase II (Bender *et al.*, 1990), cisplatin, which forms DNA adducts (Reed and Kohn, 1990), and the antibiotic MITC which can cross-link DNA and generate free radicals (Ghiorghis *et al.*, 1991). For comparison, two non-cytotoxic treatments were included, saline (the vehicle for most of the drugs) and WR-2721 (Treskes *et al.*, 1991; Green *et al.*, 1992). Most drugs were administered at or near their anticipated maximum tolerated dose and/or $LD_{10}$ (Berger *et al.*, 1991), producing approximately equitoxic treatments. Since BCNU and VP-16 might be expected to have less activity than the other agents against breast cancer cells (Henderson, 1991), these drugs were administered at higher doses. This is evidenced by the increased toxicity of these drugs relative to the others (Table IV).

The pattern of response to this selection of agents appears to reflect closely that seen in breast cancer patients. For example, breast cancers in general respond poorly to nitrosoureas (Goldman *et al.*, 1993), and this is mirrored by the poor response of MDA435/LCC6 cells to BCNU. VP-16 also did not produce long-term survivors, and this drug generally has been ineffective as a single agent in breast cancer (Henderson and Shapiro, 1991; Henderson, 1991). In contrast, DOX (Henderson and Shapiro, 1991), MITC (Garewal, 1988) and TAX (Donehower and Rowinsky, 1994) are among the most effective single agents in previously untreated breast cancer, and all of these drugs produced long-term survivors. Cisplatin is generally associated with the management of ovarian cancer, a disease in which malignant ascites also occur with a high frequency. However, it also produced long-term survivors in mice bearing the MDA435/LCC6 ascites. While not widely used in breast cancer, several studies have indicated that it may be useful as a first-line treatment, with response rates as high as 52% reported (Henderson and Shapiro, 1991). While the response rate in pretreated breast cancer patients can be lower (Henderson and Shapiro, 1991), the response in the MDA435/LCC6 ascites is consistent with the origin of the parental cells from an untreated patient.

The MDA-MB-435 cells are among the few breast cancer cell lines that reproducibly metastasise in nude mice (Price *et al.*, 1990; Meschter *et al.*, 1992). We have not studied the metastatic potential of the ascites variant, since the life span of mice bearing the ascites tumour is probably too short to allow metastases to arise. The parental cells require 8–12 weeks to produce a high incidence of lung metastases (Price *et al.*, 1990). We would expect the MDA435/LCC6 cells growing as solid tumours to exhibit a similar metastatic potential as the parental cells. Studies to determine the ability of both MDA435/LCC6 and MDA435/LCC6$^{MDR1}$ tumours to metastasise are currently in progress.

The multidrug-resistant phenotype is often accompanied by the expression of the *MDR1* gene and/or its gp170 glycoprotein product, both of which are widely detected in treated human breast tumours. Detectable gp170 expression in breast tumours increases with induction chemotherapy. (Koh *et al.*, 1992), and correlates with failure of cytotoxic chemotherapy (Goldstein *et al.*, 1989; Sanfilippo *et al.*, 1991), poor prognosis (Verrelle *et al.*, 1991), poor survival (Verrelle *et al.*, 1991; Botti *et al.*, 1993) and/or *in vitro* resistance of human breast cancer biopsies to cytotoxic drugs (Salmon *et al.*, 1989; Sanfilippo *et al.*, 1991). The precise role of MDR1/gp170 in breast cancer remains to be definitively established, but it seems highly likely that in tumours in which expression is detectable this expression contributes to the multidrugresistant phenotype. This does not exclude the possibility that gp170 expression is superimposed upon the expression of other resistance mechanisms. For example, MCF-7 cells selected *in vitro* for resistance to DOX (MCF-$7^{ADR}$) overexpress gp170 (Vickers *et al.*, 1988) and also exhibit altered manganous superoxide dismutase (Zyad *et al.*, 1994), glutathione transferase and topoisomerase II activities (Batist *et al.*, 1986; Sinha *et al.*, 1989). These cells also have lost ER and PgR expression and have acquired anti-oestrogen resistance (Vickers *et al.*, 1988). Where gp170 contributes to the multiple drug resistance phenotype, this contribution must be eliminated if chemotherapy is to be curative. Consequently, there is a need to provide models in which gp170 is the only, or at least the primary, resistance mechanism.

The MCF-$7^{ADR}$ cells have been widely used in gp170-reversing studies, but effects on cytotoxicity cannot readily be attributed solely to effects on gp170 in these cells. The tamoxifen cross-resistance in MCF-$7^{ADR}$ is the result of the loss of ER and not gp170 expression (Clarke *et al.*, 1992*b*), despite the ability of tamoxifen to reverse gp170 function, implying that tamoxifen may be a classical gp170 substrate (Ramu *et al.*, 1984; Leonessa *et al.*, 1994). Furthermore, differences in the potency of flupenthixol isomers observed in MCF-$7^{ADR}$ cells could not be confirmed in NIH 3T3 cells transfected with the MDR1 cDNA, implying a non-gp170-mediated effect on drug resistance (Ford *et al.*, 1990). While concurrently expressed drug resistance mechanisms may ultimately prove to be more clinically relevant, cells with multiple resistance mechanisms are not optimal for assessing drugs/combinations specifically selected or designed to reverse gp170-mediated resistance. To directly address this concern, we have previously generated an ER-positive model (CL 10.3) for screening endocrine-based modalities by transducing MCF-7 cells with the MDR1 cDNA (Clarke *et al.*, 1992*b*). We have already used the ER-positive model, in conjunction with the MCF-$7^{ADR}$ cells, to exclude ER-mediated events in the reversal of gp170 resistance by the anti-oestrogen tamoxifen (Leonessa *et al.*, 1994).

To generate an appropriate ER-negative gp170-resistant model for future studies, we have now transduced MDA435/LCC6 cells with a vector similar to that used to generate the CL 10.3 cells. We isolated a population of cells (MDA435/$LCC6^{MDR1}$) that express a classical MDR1 phenotype. MDA435/$LCC6^{MDR1}$, but not MDA435/LCC6 cells, express high levels of the gp170 product as determined by Western blotting with an anti-gp170 polyclonal antibody. Thus, the inserted cDNA is clearly transcribed and the resultant mRNA translated. Function of the expressed gp170 is evidenced by the significant increase in the $IC_{50}$ for DOX, VBL and TAX. The MDA435/$LCC6^{MDR1}$ cells also retain their tumorigenicity *in vivo*, indicating that they also may be useful as an *in vivo* model for screening gp170 reversing agents.

The MDA435/LCC6 and MDA435/$LCC6^{MDR1}$ cells provide a potentially novel alternative to the murine leukaemia ascites (L1210, P388) and also provide a potential solid tumour system. Thus, in conjunction with other breast cancer cell lines, these cells facilitate the *in vitro* and *in vivo* screening of new cytotoxic drugs and drug combinations. For example, the tumorigenicity of these cells is approximately 100% in untreated nude mice and rats. The ascites facilitate both assessment of long-term survivors and %ILS as *in vivo* end points, and studies into the dynamics and route of tumour spread from ascites in nude mice. The solid tumours are amenable to the estimation of anti-tumour activity on established tumours e.g. tumour growth delay (Moulder and Rockwell, 1987) and excision assays (Hill, 1987). When growing *in vitro*, MDA435/LCC6 and MDA435/$LCC6^{MDR1}$ cells can be used in any of the various anchorage-dependent and anchorage-independent growth inhibitory assays.

**Acknowledgements**
This work was supported by Public Health Service grants R01-CA58022, UO1-CA51908, P50-CA58185 and 1P30-CA51008 (R Clarke) from the National Cancer Institute. The authors thank Dr Ward D Peterson and Dr Joseph Kaplan (Children's Hospital of Michigan, Detroit, MI, USA) for the isozyme and karyotype analyses, and the veterinary pathologists at Veterinary Diagnostic Services (Baltimore, MD, USA) for performing the histological analyses of the solid tumours.

**References**

BATIST G, TUPLE A, SINHA BK, KATKI AG, MYERS CE AND COWAN KH. (1986). Overexpression of a novel anionic glutathione transferase in multidrug-resistant human breast cancer cells. *J. Biol. Chem.*, **261**, 15544–15549.

BENDER RA, HAMEL E AND HANDE KR. (1990). Plant alkaloids. In *Cancer Chemotherapy: Principles and Practice.* Chabner BA and Collins JM. (eds). pp. 253–275. JB Lippincott: Philadelphia.

BERGER DP, WINTERHALTER BR AND FIEBIG HH. (1991). Conventional chemotherapy. In *The Nude Mouse in Oncology Research.* Boven E and Winograd B. (eds). pp. 165–184. CRC Press: Boca Raton.

BOTTI G, CHIAPPETTA G, D'AIUTO G, DE ANGELIS E, DE MATTEIS A, MONTELLA M, PICONE A AND CASCIONE F. (1993). PCNA/cyclin and P-glycoprotein as prognostic factors in locally advanced breast cancer. An immunohistochemical, retrospective study. *Tumori*, **79**, 214–218.

BOYD MR. (1989). Status of the NCI preclinical antitumor drug discovery screen. *PPO Updates*, **3**, 1–12.

CAILLEAU R, YOUNG R, OLIVE M AND REEVES WJ. (1974). Breast tumor cell lines from pleural effusions. *J. Natl Cancer Inst.*, **53**, 661–674.

CAILLEAU R, OLIVE M AND CRUCIGER QVA. (1978). Long-term human breast carcinoma cell lines of metastatic origin: preliminary characterization. *In Vitro*, **14**, 911–915.

CLARKE R, BRÜNNER N, THOMPSON EW, GLANZ P, KATZ D, DICKSON RB AND LIPPMAN ME. (1989). The inter-relationships between ovarian-independent growth, antiestrogen resistance and invasiveness in the malignant progression of human breast cancer. *J. Endocrinol.*, **122**, 331–340.

CLARKE R, DICKSON RB AND LIPPMAN ME. (1992*a*). Hormonal aspects of breast cancer: growth factors, drugs and stromal interactions. *Crit. Rev. Oncol. Hematol.*, **12**, 1–23.

CLARKE R, CURRIER S, KAPLAN O, LOVELACE E, BOULAY V, GOTTESMAN MM AND DICKSON RB. (1992*b*). Effect of P-glycoprotein expression on sensitivity to hormones in MCF-7 human breast cancer cells. *J. Natl Cancer Inst.*, **84**, 1506–1512.

COLVIN M AND CHABNER BA. (1990). Alkylating agents. In *Cancer Chemotherapy: Principles and Practice*, Chabner BA and Collins JM. (eds). pp. 276–313. JB Lippincott: Philadelphia.

DE VITA, VT. (1989). Principles of chemotherapy. In *Cancer Principles and Practice of Oncology*, 3rd edn. De Vita VT, Hellman S and Rosenberg SA. (eds). pp. 276–300. JB Lippincott: Philadelphia.

DHAINAUT A, RÉGNIER G, ATASSI G, PIERRÉ A, LÉONCE S, KRAUS-BERTHIER L AND PROST J-F. (1992). New triazine derivatives as potent modulators of multidrug resistance. *J. Med. Chem.*, **35**, 2481–2496.

DONEHOWER RC AND ROWINSKY EK. (1994). Paclitaxel. *PPO Updates*, **8 (10)**, 1–16.

FORD JM, BRUGGEMANN EP, PASTAN I, GOTTESMAN MM AND HAIT WN. (1990). Cellular and biochemical characterization of thioxanthenes for reversal of multidrug resistance in human and murine cell lines. *Cancer Res.*, **50**, 1748–1756.

FRACCHIA AA, KNAPPER WH, CAREY JT AND FARROW JH. (1970). Intrapleural chemotherapy for effusion from metastatic breast cancer. *Cancer*, **26**, 626–629.

FRANDSEN TL, BOYSEN BE, JIRUS S, SPANG-THOMSEN M, DANO K, THOMPSON EW AND BRUNNER N. (1992). Experimental models for the study of human cancer cell invasion and metastasis. *Fibrinolysis*, **6**, (suppl 4), 71–76.

GAREWAL HS. (1988). Mitomycin C in the chemotherapy of advanced breast cancer. *Semin. Oncol.*, **15**, (suppl 4), 74–79.

GHIORGHIS A, TALEBIAN A AND CLARKE R. (1991). *In vitro* antineoplastic activity of N7-substituted Mitomycin C analogues (MC-77 and MC-62) against human breast cancer cell lines. *Cancer Chemother. Pharmacol.*, **29**, 290-296.

GLASS AG AND HOOVER RN. (1990). Rising incidence of breast cancer: relationship to stage and receptor status. *J. Natl Cancer Inst.*, **82**, 693-696.

GOLDMAN CA, SKINNIDER LF AND MAKSYMIUK AW. (1993). Interferon instillation for malignant pleural effusions. *Ann. Oncol.*, **4**, 141-145.

GOLDSTEIN LJ, GALSKI H, FOJO A, WILLINGHAM M, LAI S, GAZDAR A, PIRKER R, GREEN A, CRIST W, BRODEUR GM, LIEBER M, COSSMAN J, GOTTESMAN M AND PASTAN I. (1989). Expression of a multidrug resistance gene in human cancers. *J. Natl Cancer Inst.*, **81**, 116-124.

GREEN D, WRIGHT A, SCHEIN P AND CLARKE R. (1992). WR-2721 chemoprotection of doxorubicin toxicity in mice. *Proc. Am. Assoc. Cancer Res.*, **33**, 490.

HENDERSON IC. (1991). Chemotherapy for metastatic disease. In *Breast Diseases*, Harris JR, Hellman S, Henderson IC and Kinne DW. (eds). pp. 604-665. JB Lippincott: Philadelphia.

HENDERSON IC AND SHAPIRO CI. (1991). Adjuvant chemotherapy: an overview. In *Medical Management of Breast Cancer*, Powles T and Smith IE. (eds). pp. 197-215. Dunitz: London.

HILL RP. (1987). Excision assays. In *Rodent Tumor Models in Experimental Cancer Therapy*, Kallman RF. (ed.) pp. 67-75. Pergamon Press: New York.

JONAT W AND MAAT H. (1978). Some comments on the necessity of receptor determinations in human breast cancer. *Cancer Res.*, **38**, 4305-4306.

KOH EH, CHUNG HC, LEE KB, LIM HY, KIM JH, ROH JK, MIN JS, LEE KS AND KIM BS. (1992). The value of immunohistochemical detection of P-glycoprotein in breast cancer before and after induction chemotherapy. *Yonsei Med. J.*, **33**, 137-142.

LEONESSA F, BOULAY V, WRIGHT A, THOMPSON EW, BRÜNNER N AND CLARKE R. (1991). The biology of breast tumor progression: acquisition of hormone-independence and resistance to cytotoxic drugs. *Acta Oncol.*, **31**, 115-123.

LEONESSA F, JACOBSON M, BOYLE B, LIPPMAN J, McGARVEY M AND CLARKE R. (1994). The effect of tamoxifen on the multidrug resistant phenotype in human breast cancer cells: isobologram, drug accumulation and gp-170 binding studies. *Cancer Res.*, **54**, 441-447.

MESCHTER CL, CONNOLLY JM AND ROSE DP. (1992). Influence of regional location of the inoculation site and dietary fat on the pathology of MDA-MB-435 human breast cancer cell-derived tumors growing in nude mice. *Clin. Exp. Metastasis*, **10**, 167-173.

MEYERS JS, RAO BR, STEVENS SC AND WHITE WL. (1977). Low incidence of estrogen receptor in breast carcinomas with rapid rates of cellular proliferation. *Cancer*, **40**, 2290-2298.

MILLER AB AND BULBROOK RD. (1986). UICC multidisciplinary project on breast cancer: the epidemiology, aetiology and prevention of breast cancer. *Int. J. Cancer*, **37**, 173-177.

MOULDER JE AND ROCKWELL S. (1987). Comparison of tumor assay methods. In *Rodent Tumor Models in Experimental Cancer Therapy*, Kallman RF. (ed.) pp. 272-278. Pergamon Press: New York.

MYERS CE AND CHABNER BA. (1990). Anthracyclines. In *Cancer Chemotherapy: Principles and Practice*. Chabner BA and Collins JM. (eds). pp. 356-381. JB Lippincott: Philadelphia.

PASTAN I, GOTTESMAN MM, UEDA K, LOVELACE E, RUTHERFORD AV AND WILLINGHAM MC. (1988). A retrovirus carrying an MDR1 cDNA confers multidrug resistance and polarized expression of P-glycoprotein in MDCK cells. *Proc. Natl Acad. Sci. USA*, **85**, 4486-4490.

PRICE JE, POLYZOS A, ZHANG RD AND DANIELS LM. (1990). Tumorigenicity and metastasis of human breast carcinoma cell lines in nude mice. *Cancer Res.*, **50**, 717-721.

RAMU A, GLAUBIGER D AND FUKS Z. (1984). Reversal of acquired resistance to doxorubicin in P388 murine leukemia cells by tamoxifen and other triparanol analogues. *Cancer Res.*, **44**, 4392-4395.

REED E AND KOHN KW. (1990). Platinum analogues. In *Cancer Chemotherapy: Principles and Practice*. Chabner BA and Collins JM. (eds). pp. 465-490. JB Lippincott: Philadelphia.

RYGAARD K AND SPANG-THOMSEN M. (1989). 'GROWTH' - a computer program for determination of mean growth curves and calculation of response to therapy of solid tumor xenografts. In *Immune-Deficient Animals in Experimental Medicine*. Wu B and Zheng J. (eds). pp. 301-360. Karger: Basle.

SALMON SE, GROGAN TM, MILLER T, SCHEPER R AND DALTON WS. (1989). Prediction of doxorubicin resistance in vitro in myeloma, lymphoma and breast cancer by P-glycoprotein staining. *J. Natl Cancer Inst.*, **81**, 696-701.

SANFILIPPO O, RONCHI E, DE MARCO C, DI FRONZO G AND SILVESTRINI R. (1991). Expression of P-glycoprotein in breast cancer tissue and in vitro resistance to doxorubicin and vincristine. *Eur. J. Cancer*, **27**, 155-158.

SCHNEIDER J, BAK M, EFFERTH T, KAUFMANN M, MATTERN J AND VOLM M. (1989). P-glycoprotein expression in treated and untreated human breast cancer. *Br. J. Cancer*, **60**, 815-818.

SHEK LL AND GODLOPHIN W. (1989). Survival with breast cancer: the importance of estrogen receptor quantity. *Eur. J. Cancer Clin. Oncol.*, **25**, 243-250.

SICILIANO MJ, BARKER PE AND CAILLEAU R. (1979). Mutually exclusive genetic signatures of human breast tumor cell lines with a common chromosomal marker. *Cancer Res.*, **39**, 919-922.

SINGH L, WILSON AJ, BAUM M, WHIMSTER WF, BIRCH IH, JACKSON IM, LOWREY C AND PALMER MK. (1988). The relationship between histological grade, oestrogen receptor status, events and survival at 8 years in the NATO (Nolvadex) trial. *Br. J. Cancer*, **57**, 612-614.

SINHA BK, MIMNAUGH EG, RAJAGOPALAN S AND MYERS CE. (1989). Adriamycin activation and oxygen free radical formation in human breast tumor cells: protective role of glutathione peroxidase in adriamycin resistance. *Cancer Res.*, **49**, 3844-3848.

TRESKES M, HOLWERDA U, KLEIN I, PINEDO HM AND VAN DER VIJGH WJ. (1991). The chemical reactivity of the modulating agent WR2721 (ethiofos) and its main metabolites with the antitumor agents cisplatin and carboplatin. *Biochem. Pharmacol.*, **42**, 2125-2130.

VENERONI S, ZAFFARONI N, DAIDONE MG, BENINI E, VILLA R AND SILVESTRINI R. (1994). Expression of P-glycoprotein and *in vitro* or *in vivo* resistance to doxorubicin and cisplatin in breast and ovarian cancers. *Eur. J. Cancer*, **30A**, 1002-1007.

VERRELLE P, MEISSONNIER F, FONCK Y, FEILLEL V, DIONET C, KWIATKOWSKI F, PLAGNE R AND CHASSAGNE J. (1991). Clinical relevance of immunohistochemical detection of multidrug resistance P-glycoprotein in breast carcinoma. *J. Natl Cancer Inst.*, **83**, 111-116.

VICKERS PJ, DICKSON RB, SHOEMAKER R AND COWAN KH. (1988). A multidrug-resistant MCF-7 human breast cancer cell line which exhibits cross-resistance to antiestrogens and hormone independent tumor growth. *Mol. Endocrinol.*, **2**, 886-892.

ZYAD A, BERNARD J, CLARKE R, TURSZ T, BROCKHAUS M AND CHOUAIB S. (1994). Human breast cancer cross-resistance to TNF and adriamycin: relationship to MDR1, MnSOD and TNF gene expression. *Cancer Res.*, **54**, 825-831.